# Genetic architecture of spring and autumn phenology in *Salix*

**DOI:** 10.1186/1471-2229-14-31

**Published:** 2014-01-17

**Authors:** Luisa Ghelardini, Sofia Berlin, Martin Weih, Ulf Lagercrantz, Niclas Gyllenstrand, Ann Christin Rönnberg-Wästljung

**Affiliations:** 1 Department of Plant Biology, Uppsala BioCenter, Swedish University of Agricultural Sciences and Linnean Center for Plant Biology, SE-750 07 Uppsala, Sweden; 2 Present address: Institute for Plant Protection, Italian National Research Council CNR, 50019 Sesto fiorentino, Italy; 3 Department of Crop Production Ecology, Swedish University of Agricultural Sciences and Linnean Center for Plant Biology, SE-750 07 Uppsala, Sweden; 4 Department of Plant Ecology and Evolution, Evolutionary Biology Centre, Uppsala University, SE-752 36 Uppsala, Sweden

**Keywords:** Phenology, Adaptation, *Salix*, QTL, Candidate genes

## Abstract

**Background:**

In woody plants from temperate regions, adaptation to the local climate results in annual cycles of growth and dormancy, and optimal regulation of these cycles are critical for growth, long-term survival, and competitive success. In this study we have investigated the genetic background to growth phenology in a Salix pedigree by assessing genetic and phenotypic variation in growth cessation, leaf senescence and bud burst in different years and environments. A previously constructed linkage map using the same pedigree and anchored to the annotated genome of *P. trichocarpa* was improved in target regions and used for QTL analysis of the traits. The major aims in this study were to map QTLs for phenology traits in *Salix*, and to identify candidate genes in QTL hot spots through comparative mapping with the closely related *Populus trichocarpa*.

**Results:**

All traits varied significantly among genotypes and the broad-sense heritabilities ranged between 0.5 and 0.9, with the highest for leaf senescence. In total across experiment and years, 80 QTLs were detected. For individual traits, the QTLs explained together from 21.5 to 56.5% of the variation. Generally each individual QTL explained a low amount of the variation but three QTLs explained above 15% of the variation with one QTL for leaf senescence explaining 34% of the variation. The majority of the QTLs were recurrently identified across traits, years and environments. Two hotspots were identified on linkage group (LG) II and X where narrow QTLs for all traits co-localized.

**Conclusions:**

This study provides the most detailed analysis of QTL detection for phenology in *Salix* conducted so far. Several hotspot regions were found where QTLs for different traits and QTLs for the same trait but identified during different years co-localised. Many QTLs co-localised with QTLs found in poplar for similar traits that could indicate common pathways for these traits in Salicaceae. This study is an important first step in identifying QTLs and candidate genes for phenology traits in *Salix*.

## Background

Adaptation of the annual cycles of growth and dormancy to the local climate is critical for survival and competitive success of woody plants. Such local adaptation has been described in several species, and it can be seen as clines in phenology traits, including the timing of leaf emergence, leaf senescence, and growth cessation [1]. This pattern reflects the trade-off between frost tolerance and enhanced growth [2]. Cessation and initiation of growth determines the period of active stem elongation, mark the shift between frost resistant and vulnerable phases, and their timing is critical for overall biomass production, fitness and the long-term survival of species [3,4]. After height growth cessation and until leaves senesce, deciduous shrubs and trees, including willows and poplars, continue to be photosynthetically active and may accumulate considerable biomass [5-7]. In this phase, photosynthesis is critical to cold acclimation and survival in winter [8]. The timing of leaf senescence in autumn has a strong impact on nutrient retranslocation, reserve storage and the next early-season growth [5,9]. In addition, spring and autumn leaf phenology are evolutionary important traits for herbivore and pathogen resistance [10-13].

Decreasing photoperiod (day-length) is the main environmental cue inducing growth cessation and bud set in many perennial plants [14], including poplar [15]. This response to photoperiod is under strong genetic control [16-18] and is maintained when trees are moved between latitudes [19]. Temperature, alone or in combination with photoperiod, also induces growth cessation in some tree species [20-24]. Senescence and shedding of leaves are also influenced by photoperiod, often in interaction with temperature [25]. In poplars, leaf senescence is induced by shortening day-lengths but needs to be preceded by bud set. However, it is not clear to what extent senescence and bud set are under independent genetic control [26,27].

After growth cessation, dormancy is initiated, which is a prerequisite for the development of cold acclimation and freezing tolerance [28]. Dormancy release requires exposure to chilling temperatures [29]. Light does not seem to play a role in this process, but once dormancy is broken, bud burst and growth resumption are regulated by temperature, light and photoperiod. Growth resumption and bud burst depend on accumulation of temperature units over a specific threshold (thermal time) [29]. Temperature is the most important factor regulating bud burst in temperate woody plants [30], but photoperiod also plays a role in some populations and species [31,32].

Willows (*Salix*) belong together with poplars (*Populus*) to the plant family Salicaceae. Based on the fossil record the divergence of the two genera was dated to approximately 45 mya [33,34]. Willows and poplars share many characteristics such as dioecy, rapid growth and seed development, and ease with which they can be vegetatively propagated. They typically have a haploid chromosome number of 19 and similar genomes sizes of approximately 500 Mbp. Also there is strong syntheny and colinearity between willow and poplar genomes [35]. The *Salix* genus shows a remarkable phenotypic diversity ranging from small shrubs to large trees. *Salix* spp. have a global distribution in temperate and arctic regions and are adapted to a wide range of habitats [36]. Relatively high levels of genetic diversity [37] and the broad phenotypic diversity make them an excellent model system for studying evolutionary processes such as adaptation. Moreover, willows have generally rapid growth and high biomass yields and these characteristics together with ease of vegetative propagation make them economically attractive as bioenergy crops. Willows have been increasingly used in the last decades for biomass production worldwide and *Salix viminalis L*. and *S. schwerinii E*. Wolf and their hybrids are some of the most commonly used willows in the breeding programs in Europe. These two species are dioecious and outcrossing and morphologically very similar. Both are multi-stemmed shrubs with long and slender leaves and are commonly found along rivers and in other wet areas.

Growth cessation in *Salix* species is marked by the abscission of the shoot apex [25] and is controlled by photoperiod [38,39]. Large clonal variation in the time of leaf abscission has been observed in *Salix*, and delayed leaf abscission was shown to impair leaf nitrogen retranslocation and to increase nitrogen losses [6]. Extensive clonal and species variation in timing of bud burst has been observed in willow, mainly determined by differences in thermal time requirement [6,40,41]. Moderate to high heritabilities have been reported for timing of bud burst and growth cessation in different *S. viminalis* families [40,42,43].

Phenology traits have a quantitative genetic background and thus QTL mapping is a powerful method to identify genomic regions controlling phenology traits. With a reference genome one can obtain information on the genomic content of the QTL regions. With the advent of high-throughput genotyping technologies and annotated reference genomes, genetic markers in evenly spaced genes throughout the genome can be developed and genotyped for the purpose of constructing dense genome-wide linkage maps.

Here we studied the phenology of growth, including timing of bud burst, timing of cessation of elongation growth and leaf abscission in willows both in controlled and field conditions during multiple years. Our two aims were to map QTLs associated with phenology in *Salix*, and to identify candidate genes in QTL hot spots through comparative mapping with *Populus trichocarpa*. This was achieved by using a dense linkage map anchored to the annotated genome of *P. trichocarpa* and by constructing denser maps in the QTL hot spots.

## Results

### Phenotypic variation in phenology

**Figure 1:**
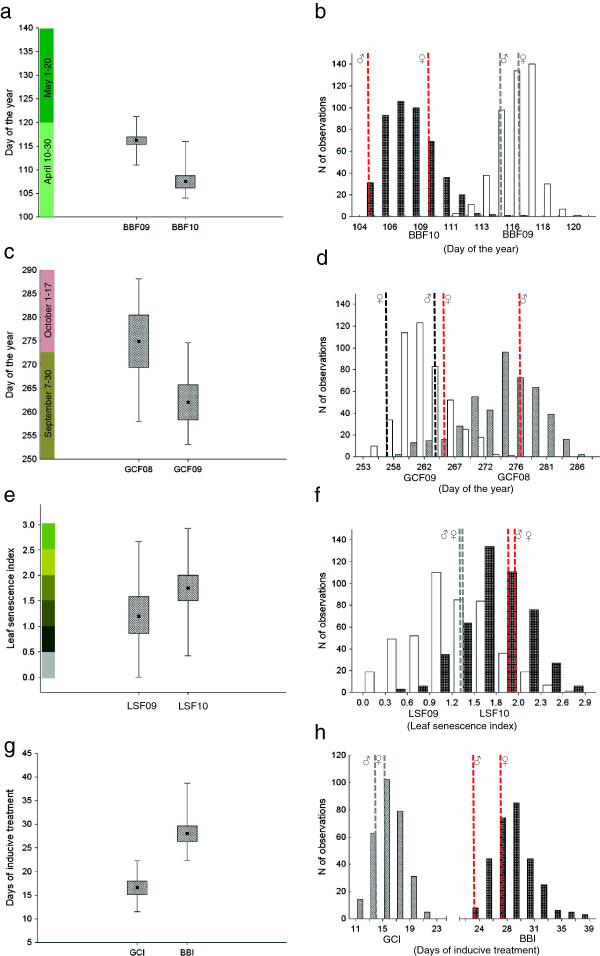
**Distributions of phenotypic mean values for the S**_**1 **_**pedigree which consists of 463 F**_**1 **_**progeny from the cross between the diploid hybrid male ‘Björn’ (*Salix viminalis *L. × *S. schwerinii *E. Wolf) and the diploid female *S. viminalis *‘78183’.** Distributions are shown for the traits bud burst **(a, b)**, growth cessation **(c, d)** and leaf senescence **(e, f)** in field conditions during several years and indoors **(g, h)**.

In the S_1_ pedigree, planted in an experimental field in Pustnäs, south of Uppsala (59°48′ N, 17°39′E, 25 m), the mean date for bud burst was 20^th^ of April (day of the year (DOY) 112, Figure 1), the date of apex abscission (growth cessation) was 24^th^ of September (DOY 268, Figure 1), and about 25% yellow leaves were left on the plants at the end of October (leaf senescence index LSI = 1.5; DOY 304, Figure 1). Year-to-year variation was significant for all traits (Table 1, Figure 1), and genotype ranking significantly changed across years (Table 1, significant genotype × year interaction) indicating different responses to seasonal variation between genotypes. In the indoor experiment, elongation growth ceased on average 2 weeks after progressive reduction of the photoperiod. After nine weeks of artificial winter in the indoor experiment, bud burst occurred after 4 weeks of increased temperature and day length, corresponding to 336 day degrees > 0°C (Figure 1g, h).

**Table 1:** ANOVA for year and block × genotype interaction on phenology traits in the field

	**Bud burst (BBF)**		**Growth cessation (GCF)**		**Leaf senescence (LSF)**	
	**F**	**df**_**1**_**/df**_**2**_	**F**	**df**_**1**_**/df**_**2**_	**F**	**df**_**1**_**/df**_**2**_
Genotype effect	2.08***	309/309	3.10***	139/139	5.36***	452/452
Year effect	6405.57***	1/309	1281.29***	1/139	908.32***	1/452
Genotype × year	1.95***	309/3100	1.73***	139/1400	2.47***	452/4530
						
Genotype effect^a^	4.39***	309/1545	5.57***	139/695	13.99***	452/2260
Block effect^a^	34.60***	5/1545	8.16***	5/695	66.03***	5/2260
Genotype × block^a^	0.12^ns^	1545/1860	0.26^ns^	695/840	0.47^ns^	2260/2718

^a^ANOVA performed on pooled data from 2 years. ***, Significant at the 0.01% level, ns= non significant.

In the progeny, all traits varied significantly among genotypes (Table 1, Figure 1). The variation of genotypic means for bud burst was smaller in the field than indoors (11 vs. 17 days, Figure 1b, h). A large part of the phenotypic variation for all the traits and in all experiments was due to genetic factors as shown by relatively high broad-sense heritabilities (0.5–0.9) (Table 2). The highest broad-sense heritability was found for leaf senescence. There were strong block effects (Table 1), but all genotypes were similarly affected in the field (Table 1, no genotype × block interaction) and indoors (among-blocks correlation r=0.5–0.7, p<0.001). Therefore, QTL analyses were performed on unadjusted clonal means, which should be a good estimate of the average behaviour of a genotype.

**Table 2:** Traits description, clonal mean heritability, and ANOVA results from variance components analysis

**Trait**	**Year**	**Name**	**Unit**	**H**^**2**^	**F**_**(df1/df2)**_**Genotype effect**	**F**_**(df1/df2)**_**Block effect**
Growth chamber experiment						
Bud burst	2008	BBI08	Days	0.78	4.49**_(293/586)_	12.25**_2/586_
Growth cessation	2008	GCI08	Days	0.65	2.86**_(293/586)_	186.3**_(2/586)_
Field experiment						
Bud burst	2009	BBF09	DOY	0.52	2.10**_(461/2060)_	4.10^ns^_(5/2060)_
Bud burst	2010	BBF10	DOY	0.81	5.30**_(461/2415)_	136.4^ns^_(5/2415)_
Growth cessation	2008	GCF08	DOY	0.77	4.30**_(461/2157)_	11.64**_(5/2157)_
Growth cessation	2009	GCF09	DOY	0.71	3.47**_(461/2099)_	21.84**_(5/2099)_
Leaf senescence	2009	LSF09	Senescence %	0.92	12.91**_(461/2415_	18.73**_(5/2415)_
Leaf senescence	2010	LSF10	Senescence %	0.83	5.86**_(461/2296)_	91.77**_(5/2296)_

H^2^,clonal mean heritability, **p<0,01, ^ns^non significant.

Bud burst and growth cessation showed a significant correlation only in 2008. Positive but weak correlations between the field and indoor were found both for bud burst and growth cessation. In the field, bud burst, growth cessation, and leaf senescence were each positively correlated between years (Table 3, plots in Additional file 1: Figure S1).

**Table 3:** Pearson correlation coefficients between all phenology traits across years and experiments

	**LSF09**^**1**^	**LSF10**^**1**^	**BBF09**^**1**^	**BBF10**^**1**^	**GCF09**^**1**^	**GCF08**^**1**^	**BBI**^**2**^	**GCI**^**2**^
LSF09		+0.70	-0.01	-0.09	+0.40	+0.37	+0.14	+0.20
		p<0.001	p=0.82	p=0.057	p<0.001	p<0.001	p=0.02	p=0.001
LSF10			+0.01	-0.02	+0.36	+0.35	+0.12	+0.14
			p=0.76	p=0.65	p<0.001	p<0.001	p=0.049	p=0.02
BBF09				+0.35	-0.001	+0.07	+0.19	+0.05
				p<0.001	p=0.98	p=0.87	p=0.001	p=0.39
BBF10					-0.04	-0.04	+0.30	+0.11
					p=0.37	p=0.44	p<0.001	p=0.07
GCF09						+0.59	+0.06	+0.15
						p<0.001	p=0.29	p=0.01
GCF08							+0.16	+0.19
							P=0.007	p=0.001
BBI								+0.02
								p=0.68

^1^N=462, ^2^N=294 (see the Methods section for details). Correlations are based on genotypic means (3–6 individual plants per clone).

### QTL mapping

**Figure 2:**
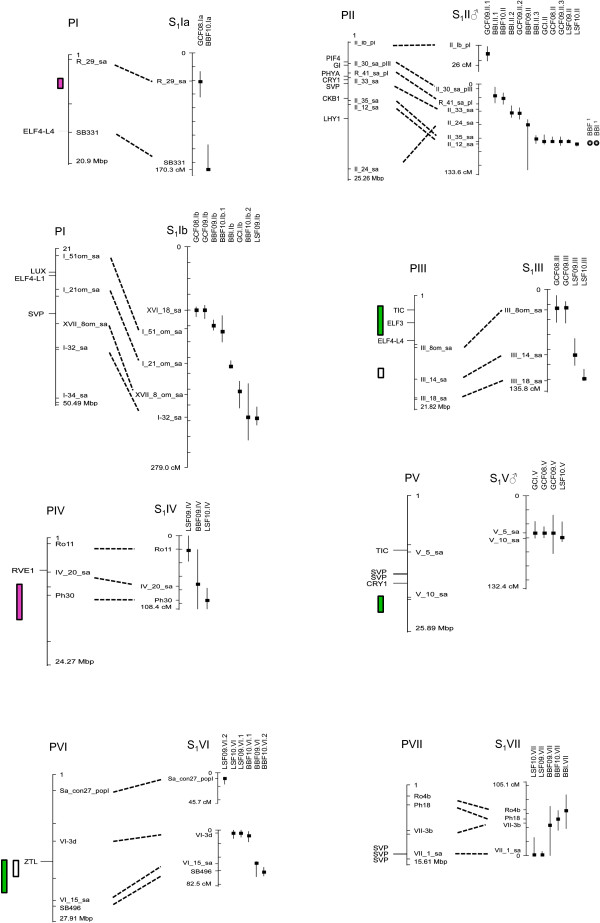
**Location of spring and autumn phenology QTLs on the consensus genetic linkage map from the S**_**1 **_**pedigree on LG I to VII.** The peak position of the local LOD (P in Table 4) is marked as a squared symbol and a support interval of 1.5-unit drop off on either side of the LOD peak is reported for each QTL. QTLs are named as in Table 4. Candidate genes and their position in the corresponding *Populus* chromosome are indicated. Grey circles on LG II: Bud burst QTLs in field (BBF) and indoor (BBI) conditions from [42] (same mapping pedigree, tentative positions based on common AFLPs). QTLs for phenology traits in *Populus* (taken from literature) are indicated. Red boxes: Selected QTLs associated with bud set (several stages and sub-processes) in *Populus nigra* from Table six in [50]. White boxes: Bud set or bud flush QTLs from [48] at tentative positions, based on their distance from a SSR marker (Chr III) or phenology candidate genes (Chr VI) in the original publication (as in [18]) Green boxes: Bud set robust quantitative trait loci (QTL) regions in [18].

**Figure 3:**
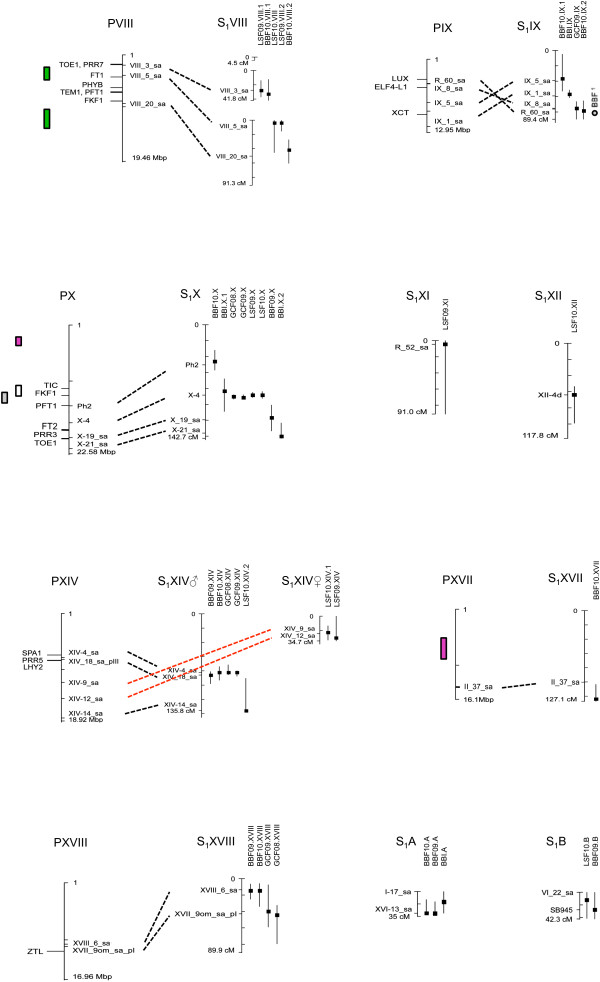
**Location of spring and autumn phenology QTLs on the consensus genetic linkage map from the S**_**1 **_**pedigree on LG VIII to XIX.** The peak position of the local LOD (P in Table 4) is marked as a squared symbol and a support interval of 1.5-unit drop off on either side of the LOD peak is reported for each QTL. QTLs are named as in Table 4. Candidate genes and their position in the corresponding *Populus* chromosome are indicated. Grey circle on LG IX: Bud burst QTLs in field (BBF) conditions from [42] (same mapping pedigree, tentative positions based on common AFLPs). QTLs for phenology traits in *Populus* (taken from literature) are indicated. Red boxes: Selected QTLs associated with bud set (several stages and sub-processes) in *Populus nigra* from Table six in [50]. White boxes & Grey boxes: Bud set or bud flush QTLs from [48] at tentative positions, based on their distance from phenology candidate genes (Chr X) in the original publication (as in [18]) Green boxes: Bud set robust quantitative trait loci (QTL) regions in [18].

In total across experiments and years, 80 QTLs were detected (Table 4, Figures 2 and 3). QTLs were named by the trait (BB = bud burst, GC = growth cessation, LS = leaf senescence), environment (I = indoors or F = field), year of assessment, linkage group where it was located, and if multiple QTLs were mapped to the same group, by an ordering number. On average about 40% (21.5–56.5% depending on the trait) of the variation among genotypic means was explained by a general model including all the QTLs that remained significant after backward selection. Individual QTLs generally explained a small proportion of the variance of the genotypic means. Eleven QTLs contributed to 7% or more of the trait variation, 3 QTLs explained more than 15%, and 1 QTL explained up to 34% of the variation (Table 4).

**Table 4:** Results of the QTL mapping procedure for each phenology trait

**QTL**	**LG**	**Marker**^**a**^	**P (cM)**^**b**^	**SI (cM)**^**c**^	**LOD**	**α**^**d**^	**PEV**^**e**^
*Bud burst in indoor conditions (BBI)*							
BBI.Ib	Ib	I_21om_sa (L29gr.164f)	150.9	9.5	7.86	0.001 gw	3.5
BBI.II.1	II♂-2	II_30_sa_pIII	16.9	23.5	4.54	0.005 cw	7.5
BBI.II.2	II♂-2	II_33_sa	42.1	17.5	4.22	0.007 cw	excl
BBI.II.3	II♂-2	II_35_sa (L12b.203f)	80.6	12	6.20	0.006 gw	7.1
BBI.VII	VII	Ro4b	64.4	48.5	3.04	0.032 cw	excl
BBI.IX	IX	IX_1_sa	56.6	10	3.60	0.010 cw	3.2
BBI.X.1	X	X-4	83.7	41	3.55	0.015 cw	excl
BBI.X.2	X	X-21_sa (L3gr.227f)	140.2	19.2	3.36	0.023 cw	2.8
BBI.A	A	XVI-13_sa	17.0	35	3.95	0.001 cw	6.2
BBI total sum of explained variance							30.3
*Bud burst in field conditions year 2009 (BBF09)*							
BBF09.Ib	Ib	XVI_12_om_sa_pI	99.5	13	6.65	0.001 gw	10.3
BBF09.II	II♂-2	II_24_sa	59.9	74.5	2.78	0.038 cw	2.0
BBF09.IV	IV	IV_20_sa (L2gr.226f)	71.1	87.9	3.00	0.028 cw	excl
BBF09.VI	VI-1	VI_15_sa (L13y.371)	48.6	22.5	5.24	0.011 gw	3.7
BBF09.VII	VII	VII-3b	43.6	70.5	2.78	0.043 cw	excl
BBF09.X	X	X_19_sa	116.7	32.5	4.19	0.003 cw	1.4
BBF09.XIV	XIV♂	XIV_18_sa_pIII	85.9	17	5.89	0.003 gw	4.4
BBF09.XVIII	XVIII	XVIII_6_sa	14.6	18.5	3.25	0.018 cw	4.5
BBF09.A	A	XVI_13_sa	34.9	18.5	2.77	0.001 cw	excl
BBF09.B	B	SB945	27.7	42.3	2.28	0.049 cw	1.3
BBF09 total sum of explained variance							27.6
*Bud burst in field conditions year 2010 (BBF10)*							
BBF10.Ia	Ia	SB331	170.3	36.3	4.60	0.005 cw	1.9
BBF10.Ib.1	Ib	I_51om_sa	107.2	28	3.78	0.022 cw	excl
BBF10.Ib.2	Ib	I-32_sa	215.5	71.5	3.63	0.026 cw	4.2
BBF10.II	II♂-2	R_41_sa_pI	20.9	17	13.32	0.001 gw	14.1
BBF10.VI.1	VI-1	VI-3d	8.3	15.5	7.96	0.005 gw	7.3
BBF10.VI.2	VI-1	SB496	61.8	13	4.04	0.005 cw	excl
BBF10.VII	VII	Ph18	52.2	28.5	4.78	0.002 cw	1.0
BBF10.VIII.1	VIII-2	VIII_3_sa	32.7	29.3	4.75	0.001 cw	5.6
BBF10.VIII.2	VIII-3	VIII_20_sa_pI	41.4	33	6.72	0.025 gw	3.4
BBF10.IX.1	IX	IX_5_sa	36.9	47.5	3.79	0.008 cw	2.1
BBF10.IX.2	IX	R_60_sa_pI	78.2	23.9	3.73	0.011 cw	3.5
BBF10.X	X	Ph2	45.9	25	4.92	0.002 cw	3.2
BBF10.XIV	XIV♂	XIV-4_sa	82.3	19	3.38	0.019 cw	excl
BBF10.XVII	XVII	II_37_sa_pI	123.8	23.5	3.19	0.041 cw	excl
BBF10.XVIII	XVIII	XVIII_6_sa	14.6	28	4.34	0.002 cw	3.0
BBF10.A	A	XVI-13_sa	35.0	22	3.05	0.001 cw	3.9
BBF10 total sum of explained variance							53.2
*Growth Cessation in indoor conditions (GCI)*							
GCI.Ib	Ib	XVII_8om_sa	182.8	34	3.93	0.015 cw	5.4
GCI.II	II♂-2	II_35_sa	84.8	14	4.70	0.006 cw	4.6
GCI.V	V♂	V-5_sa	52.8	24	5.22	0.028 gw	11.5
GCI total sum of explained variance							21.5
*Growth cessation in field conditions year 2008 (GCF08)*							
GCF08.Ia	Ia	R_29_sa	42.0	37.5	3.38	0.043 cw	2.3
GCF08.Ib	Ib	XVI_18_sa	80.1	12.5	7.28	0.001 cw	6.1
GCF08.II	II♂-2	II_35_sa	84.8	8	10.54	0.002 gw	16.7
GCF08.III	III	III_8om_sa	24.8	36	2.91	0.045 cw	1.7
GCF08.V	V♂	V-5_sa	52.8	16	5.93	0.001 cw	5.5
GCF08.X	X	X-4	90.4	5.5	16.31	0.001 gw	2.3
GCF08.XIV	XIV♂	XIV-4_sa	82.3	14	4.42	0.005 cw	2.8
GCF08.XVIII	XVIII	XVII_9om_sa_pI	43.9	47	2.83	0.043 cw	3.5
GCF08 total sum of explained variance							40.9
*Growth cessation in field conditions year 2009 (GCF09)*							
GCF09.Ib	Ib	XVI_18_sa	80.2	14.5	4.75	0.004 cw	4.9
GCF09.II.1	II♂-1	II-Ib_pI	11.0	26	4.02	0.001 cw	7.0
GCF09.II.2	II♂-2	II_33_sa	43.1	17	4.82	0.004 cw	2.4
GCF09.II.3	II♂-2	II_35_sa	84.8	13	6.15	0.001 cw	6.2
GCF09.III	III	III-4_sa	23.8	29	2.95	0.047 cw	3.2
GCF09.V	V♂	V_5_sa	52.8	55	3.15	0.032 cw	1.2
GCF09.IX	IX	IX_8_sa	75.2	22.9	3.82	0.007 cw	4.2
GCF09.X	X	X-4	91.4	5.5	8.55	0.002 gw	10.7
GCF09.XIV	XIV♂	XIV-4_sa	82.3	7.5	5.50	0.001 cw	1.4
GCF09.XVIII	XVIII	XVIII_8_sa	39.7	51.5	2.88	0.042 cw	2.1
GCF09.Ib × GCF09.XVIII							5.8
GCF total sum of explained variance							49.1
*Leaf senescence in field conditions year 2009 (LSF09)*							
LSF09.Ib	Ib	SB265	219.5	23	4.34	0.007 cw	1.6
LSF09.II	II♂-2	II_35_sa	84.8	4	32.11	0.001 gw	34.2
LSF09.III	III	III-14_sa	87.1	36	3.57	0.018 cw	excl
LSF09.IV	IV	Ro11	21.6	38	3.0	0.030 cw	excl
LSF09.VI.1	VI-1	VI-3d	4.3	10.5	3.32	0.023 cw	1.2
LSF09.VI.2	VI-2	Sa_con27_PopI	8.4	10.5	8.11	0.001 gw	3.7
LSF09.VII	VII	VII_1_sa	1.0	6	6.18	0.001 gw	4.3
LSF09.VIII.1	VIII-2	VIII_3_sa	27.7	23	3.30	0.005 cw	1.6
LSF09.VIII.2	VIII-3	VIII_5_sa	4.0	15.5	7.55	0.001 gw	5.6
LSF09.X	X	X-4	88.4	6.5	20.49	0.001 gw	2.2
LSF09.XI	XI	R_52_sa	4.5	91	3.14	0.021 cw	1.1
LSF09.XIV	XIV ♀	XIV-12_sa	30.0	34.7	2.47	0.030 cw	1.0
LSF09 total sum of explained variance							56.5
*Leaf senescence in field conditions year 2010 (LSF 2010)*							
LSF10.II	II♂-2	II-12_sa	89.6	5.5	16.89	0.001gw	19.3
LSF10.III	III	III-18_sa	118.6	15.5	6.35	0.003 gw	6.0
LSF10.IV	IV	Ph30	95.3	30.9	2.88	0.034 cw	2.3
LSF10.V	V♂	V_10_sa	59.4	28.5	3.64	0.005 cw	5.0
LSF10.VI	VI-1	VI-3d	4.3	12.5	2.94	0.049 cw	1.8
LSF10.VII	VII	VII_1_sa	1.0	26	2.73	0.047 cw	1.4
LSF10.VIII	VIII-3	VIII_5_sa	4.0	45.5	2.91	0.002 cw	1.8
LSF10.X	X	X-4	88.4	8.5	10.49	0.001 gw	1.8
LSF10.XII	XII	XII-4d	63.7	45.5	3.22	0.016 cw	1.2
LSF10.XIV.1	XIV♀	XIV-9_sa	22.8	20	6.73	0.001 gw	3.2
LSF10.XIV.2	XIV♂	XIV-14_sa	135.8	45.3	4.60	0.030 gw	excl
LSF10.B	B	VI_22_sa	12.0	41.5	4.51	0.037 gw	excl
LSF10 total sum of explained variance							43.8

^a^The closest SNP marker to the QTL peak position, also used in multiple QTL mapping, and in mixed model and variance components analyses. When the closest marker to peak position was not a SNP, the AFLP marker is reported in brackets. ^b^QTL position on the S_1_ linkage map [35]. ^c^Heuristic support interval determined as a 1.5-unit drop off on either side of the local LOD score peak. ^d^Probability for the null hypothesis of no QTL obtained after 5,000 permutations. ^e^Percentage of variance explained by the QTL or by the QTL × QTL interaction estimated by variance components analysis. gw Genome-wide, significant QTL. cw Chromosome-wide, suggestive QTL. excl QTL excluded from the complete model following a backward selection procedure at a test level of 0.05 (see Methods for details).

### QTLs for bud burst

For bud burst, 9 QTLs were identified indoors and 26 in the field during two years of assessments. Each of the QTLs included in the final model explained 1.0 to 14.1% of the variation in genotypic means (Table 4). The final model including all QTLs explained from 27.6% to 53.2% of the observed variance depending on environment and year of assessment (Table 4). No significant QTL by QTL interaction was detected for bud burst.

### QTLs for growth cessation and leaf senescence

For growth cessation, 3 QTLs were identified indoors and 18 in the field during two years of assessments. The contribution of each QTL to the phenotypic variance was low to moderate (1.2% - 16.7%) but all QTLs together explained from 21.5 to 49.1% of the trait variation (Table 4). For leaf senescence, 24 QTLs were identified across the two years of assessment. The final model (10 QTLs) explained 56.5% of the trait variation in 2009 and 43.8% in 2010. The majority of QTLs made a low contribution to the total variance (1.0 – 6.0%) with the exception of LSF09.II and LSF10.II, which explained 34.2% and 19.3% respectively (Table 4).

### Comparison of QTL positions among years, environments and traits

QTLs were considered to co-localize when their peak positions were less than 10 cM apart. Twenty-eight QTLs were identified only once while at 20 other positions at least 2 QTLs were mapped. Among the 20 regions where co-localizing QTLs were found, 11 included 2 QTLs for the same trait identified in 2 years or both environments, 4 included 2 QTLs for different traits, and 5 included 3 to 6 QTLs affecting different phenology traits (Table 4, Figures 2 and 3).

For bud burst in the field, in five cases QTLs appeared more than once at the same map position comparing the two years (Figures 2 and 3). In contrast, for growth cessation in the field, most of the QTLs mapped to the same position both years (Figures 2 and 3). Six genomic regions were detected for leaf senescence where QTL appeared both years (Figures 2 and 3).

In only one case were QTLs involved in bud burst found at the same map location when comparing indoors and the field conditions (Figure 2). One QTL for growth cessation was only identified indoors while another two co-localized with QTLs identified in the field (Figures 2).

### Improved mapping of two QTL hot spots for phenology on LG II and LG X

**Figure 4:**
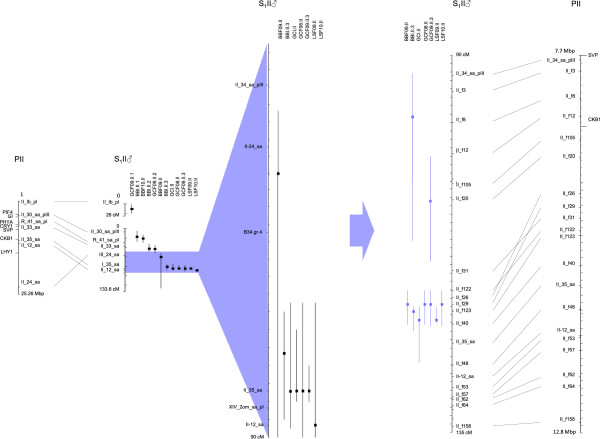
**QTL hotspots on LG II for the mapped traits (BB = bud burst, GC = growth cessation and LS = leaf senescence) and alignment to the poplar (*P. trichocarpa*) physical map.** Homologous markers are connected with dotted lines. The positions of candidate genes involved in the photoperiodic pathway and circadian clock are shown on the poplar chromosomes. The regions with increased marker density are shown to the right of the arrows.

**Figure 5:**
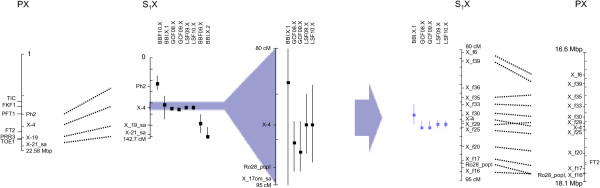
**QTL hotspots on LG X for the mapped traits (BB = bud burst, GC = growth cessation and LS = leaf senescence) and alignment to the poplar (*P. trichocarpa*) physical map.** Homologous markers are connected with dotted lines. The positions of candidate genes involved in the photoperiodic pathway and circadian clock are shown on the poplar chromosomes. The regions with increased marker density are shown to the right of the arrows.

The QTLs on LG II and X for bud burst, growth cessation and leaf senescence were confirmed with the new denser linkage map. A comparison of the QTL mapping with the linkage map from Berlin *et al*. [35] and the new maps is illustrated in Figures 4 and 5. The peak position of the QTLs was changed for all traits on LG II while only to some extent for the growth cessation traits on LG X. The 1.5 LOD intervals of the QTLs were considerable shortened with the new linkage maps (Figures 4 and 5). For the traits BBI and GCF09 the new analysis identified two QTLs on LG II for each trait instead of one as in the original analysis (Figure 4).

### Positional information of QTLs and identification of candidate genes

We predicted the genomic interval for each QTL and summed all intervals for the traits, BBF09, BBF10, BBI, GCF08, GCF09, GCI, LSF09 and LSF10. This was possible by anchoring the SNP markers to the *P. trichocarpa* genome. The total genomic intervals varied between 15.1 and 69.0 Mbp for the traits (interval for each QTL see Additional file 2: Table S1) and the total number of gene models ranged from 565 to 6,604. The largest number of gene models was found for BB10, however, 39% is due to one QTL with a large 1.5 LOD interval on LG I. Since some QTLs covered the same genomic interval, some gene models appeared more than once, we therefore estimated the number of unique gene models for BB, GC and LSF to 9,633, 4,355 and 4,815 respectively (Table 5). The total number of gene models in the intervals as well as the putative candidate genes for growth cessation are presented in Table 5. Several putative candidate genes were identified among these gene models (Figures 2 and 3; Additional file 3: Table S2). Candidate genes in the QTL intervals include photoreceptors as well as several circadian clock genes and downstream components. Among photoreceptors both cryptochrome and phytochrome genes were identified (Figures 2 and 3; Additional file 3: Table S2). The phytochrome gene PHYB2 was located in a narrow range between two QTLs on LG X and should not be ruled out as a potential gene influencing growth control in willows. Gene models within QTLs also included several core circadian clock genes such as *LATE HYPOCOTYL (LHY), PSEUDO-RESPONSE REGULATOR 7 (PRR7), LUX ARRHYTHMO (LUX), EARLY FLOWERING 3 (ELF3), ZEITLUPE (ZTL)* and *GIGANTEA (GI)* (Figures 2 and 3; Additional file 3: Table S2) [44]. Interestingly the FT2 gene is located in the proximity of the fine mapped region on LG X (Figure 5). Noteworthy is that PtFT2 [45] and PttLHY [46] are involved in the control of growth cycle in *Populus*.

**Table 5:** Genomic intervals and number of gene models in QTL regions

**Trait**	**No. of QTLs with positional information**	**Total genomic interval of QTLs (Mbp)**	**Range (Mbp)**	**No. of gene models**
BBI	8	17.8	0.3 -7.6	1,841
BBF09	7	30.0	0.5-13.3	2,676
BBF10	17	69.0	1.4 -26.7	6,604
GCI	5	15.1	1.4-11.8	565
GCF08	10	33.8	0.04-11.8	2,912
GCF09	11	29.6	0.04-11.8	2,836
LSF09	4	18.8	0.04-4.3	2,106
LSF10	5	38.1	0.04-11.8	3,412

## Discussion

The present study explores the genetic architecture of growth phenology in a pedigree between *S. viminalis* and *S. schwerinii*. Using a linkage map based on several hundreds of SNP markers, we identified QTLs for bud burst, growth cessation and leaf senescence in different years and environments. In the field, the QTLs explained together more than 40% of the variation in each trait. Several regions were identified where many QTLs co-localized for different traits or for the same trait across years. Since the SNPs were developed using the *P. trichocarpa* genome as a template, we obtained positional information for the *Salix* QTLs, projected them on the poplar genome, and identified the corresponding genomic intervals. The results suggest that some QTLs might be homologous to *Populus* QTLs. Moreover, in the projected QTL intervals we could identify putative candidate genes for the traits.

### Phenotypic variation

All phenotypic traits varied among the progeny. A large part of the variation was imputable to genetic factors, which confirms moderate to high broad sense heritabilities for bud burst and growth cessation in *Salix* species [42,43]. Although heritability estimates are known to be environment and population specific, it is now well established that bud phenology is under strong genetic control in Salicaceae [16,18,47-51].

The behaviour of the progenies changed between controlled and field conditions. Growth cessation in short days indoors and apex abscission in the field displayed particularly weak correlations. One possible explanation to this discrepancy could be that the variation observed indoors only reflects a photoperiodic response, while the variation observed in the field could reflect effects of other environmental factors. In fact, short days alone can induce apical growth cessation both in seedlings and rooted cuttings of several *Salix* species, while in field conditions, apical growth cessation does not seem to be regulated by photoperiod, but other factors seem to be involved [38,52]. In *S. viminalis*, the coincidence of growth rate decline and tip senescence with development of low leaf water potential in summer suggests an effect by water stress [52].

**Figure 6:**
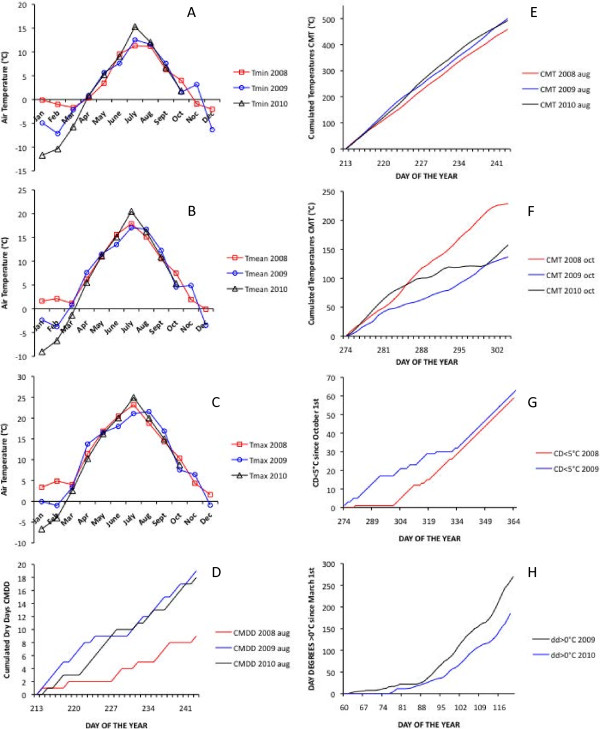
**Meteorological variables at the field site in Ultuna (Sweden) during the three years of study and in relevant periods of time.** Monthly minimum **(a)**, mean **(b)**, and maximum **(c)** temperatures; number of days with no rain (cumulated dry days, CMDD) since August 1st **(d)**; cumulative sums of daily mean temperatures (CMT,°C) during August **(e)** and October **(f)**; number of chill days (CD) with mean temperature below 5°C **(g)**; cumulated mean temperatures above 0°C (day degrees>0°C) from March 1st to bud burst **(h)**.

The timing of phenological events changed across years, as shown by Weih [6] for other *Salix* clones. In addition, the ranking of the clones changed from one year to another, which indicates a plastic behaviour in the family. This might represent a differential response among the progenies to the seasonal differences in rainfall and temperature observed between years (Figure 6). Plasticity is crucial for a species to respond to the demands of a changing environment. An increasing number of studies indicate that temperature and stress factors may variably interact with photoperiod in controlling the timing of phenological events in woody species, including willow and poplar [20,53,54]. In *S. viminalis*, the effects of photoperiod seem to be superimposed on those of water stress in controlling apical growth cessation [52]. A drier and warmer summer, i.e. higher temperature sum, higher maximum temperatures and a greater number of dry days (Figure 6), might indeed be partly responsible for the markedly earlier growth cessation observed in 2009 in this study. Combined effects of photoperiod and temperature could be also responsible for the inter-annual variability observed for leaf senescence in our willow pedigree. In *Populus*, yellowing of the leaves is initiated by a photoperiodic stimulus, but the progression of senescence is accelerated under low temperature [26]. Moreover in *Populus*, senescence seems to be faster in trees that have a late onset of senescence, independently on the effect of temperature. There is no data about regulation of seasonal leaf senescence in *Salix*. However, if we hypothesize a similar regulation as in poplar, the smaller total phenotypic variation (the full range of phenotypic variation in leaf abscission between individual plants was 40 - 100% in 2009, while 10 - 100% in 2010; not shown) and the more advanced stage of senescence observed in 2009 compared to 2010 could both be explained as an effect of lower temperatures (means, minima and maxima) in October 2009 (Figure 6a, b, c).

### QTLs for phenology traits

The phenology traits were typically quantitative. Several QTLs were found for each trait of which the majority explained less than 10% of the total variation. This was not surprising and seems to be a common feature of phenological traits among many species in the Salicaceae plant family [18,50,51]. Indeed, the regulation of phenology is quite complex and involves different pathways [14,55-57]. When estimating the total phenotypic variation of all QTLs for each trait we found particularly high values for leaf senescence, e.g. LSF10 explained as much as 56.5% of the total variation. The total phenotypic variation explained by the QTLs for the other traits was less strong but still substantial.

#### Colocalization of QTLs

At the level of resolution attained, there is considerably coincidence in map positions among a number of the QTLs. The most striking instances are found on LG II and X, where QTLs with narrow confidence intervals for BB, GC and LS consistently mapped across years and environments are located at similar positions. The QTLs on LG II also co-locate with bud burst QTLs found by Tsarouhas *et al*. [42] where they used a subset of the same willow pedigree and a different linkage map with some common markers (Figures 2 and 3). Fine mapping with additional markers on LG II and LG X further supported that QTLs for all three traits might represent the same locus. Similarly, on LG V, QTLs for growth cessation and leaf senescence co-localized, on LG VI, QTLs for leaf senescence and bud burst co-localized and on LG XIV QTLs for bud burst and growth cessation were found at a similar genomic position. This suggests that pleiotropic effects of individual QTLs on several traits could result from shared components of the pathways controlling the different traits. This is perhaps not unexpected given that both bud burst and growth cessation are both controlled jointly by photoperiod and temperature. The time of leaf senescence has been shown to be an important trait for seasonal acclimation as well as for biomass production in willows [6]. It generally occurs well after growth cessation, but data from *Populus tremula* suggest that also the onset of leaf senescence is under photoperiodic control although it might respond to a different photoperiod than bud set [26]. The frequent co-localization of QTLs for growth cessation and leaf senescence suggests that genes controlling growth cessation may to a large extent also affect the timing of leaf senescence. Furthermore, the common QTLs on some of the linkage groups suggest that these locations indeed contain genes with a central role in controlling seasonal growth in willows.

#### Comparison of QTL regions between poplar and willows

When comparing the willow QTLs with QTLs found in poplar many QTLs were found in similar genomic positions, which suggests common mechanisms controlling these traits in willows and poplar. Rohde *et al*. [18] mapped six narrow QTLs for bud set in poplar to LG III, V, VI and two on VIII and XIII, and we find QTLs for growth cessation or leaf senescence at five of these positions (Figures 2 and 3). Fabbrini et al. [50] identified several QTLs for bud set in *P. nigra* of which those on LG I and IV overlap with QTLs for growth cessation and leaf senescence in willows. Frewen et al. [48] mapped three QTLs in poplar for bud set to LG III, VI and X of which those on III and VI was also identified in willows. There are also some major differences as for example the QTLs on LG II and IX in willows are missing in poplars. However the presence or absence of a QTL for a trait depends on several factors such as accuracy of the phenotyping, environmental factors and perhaps most importantly on whether or not the trait or gene is variable in the mapping population under study. In this study we have used one mapping population from a back cross between two species and how general these results are should be validated in another genetic background.

### Identification of positional candidate genes

Comparative mapping with poplars represents a first step to further dissect the genetic basis of phenological variation in willows and ultimately identify genes or alleles responsible for the trait variation that we observe. Our understanding of the physiology and biochemistry of the traits of interest very much define the success of this approach as it relies on previous identification of genes potentially involved in the control of the traits as well as on genome conservation between poplars and willows. We have previously shown that overall gene order is conserved between willows and poplars except for few large-scale chromosomal rearrangements [35], which justify anchoring the *Salix* QTLs to the physical map of poplar. We started by estimating the number of genes within the QTLs for each trait and found as many as 9,633 genes for bud burst and about half the number of genes for growth cessation and leaf senescence. Functionally characterized genes for phenology are still scarce in poplar apart from two examples. The poplar gene *PtFT2* located on LG X that is known to be involved in growth cessation [45], was located close to the fine mapped region. *LHY*-genes have recently been shown to be involved in both growth cessation and budburst [46] and *LHY1* was located close to the QTL hot spot region on LG II and *LHY2* was located in a cluster of QTLs for bud burst and growth cessation on LG XIV. The fine mapped region on LG II contains a *SVP* homolog. In perennial species *SVP* genes have been shown to be involved in the growth cycle typically with high expression levels during bud dormancy [58-61]. The above-mentioned genes are strong candidate genes for growth cessation and bud burst that warrant further investigation in willows.

## Conclusion

We identified substantial variation in all traits in the pedigree and all traits were associated with many QTLs that each explained less than 10% of the variation, a typical pattern of quantitative characters. In total, we identified 80 QTLs, of which some were clustered in hotspots where QTLs for the different traits co-localised. Two such hotspots on LG II and X were further investigated by the construction of denser linkage maps in these regions, an effort that greatly reduced the QTL intervals (and number of gene models). Some QTLs appear to co-localize with those found in poplars, which could indicate common pathways for these traits in Salicaceae. This study is an important first step in identifying QTLs and candidate genes for phenology traits in *Salix* but further work is needed *e.g*. to confirm the QTLs in other genetic backgrounds, further fine mapping and functional studies, to verify candidate genes.

## Methods

### Plant material, experimental design and phenotyping

The S_1_ pedigree consists of 463 F_1_ progeny from the cross between the diploid hybrid male ‘Björn’ (*Salix viminalis* L. × *S. schwerinii* E. Wolf) and the diploid female *S. viminalis* ‘78183’ originating from southern Sweden. The *S. schwerinii* parent (79069) of Björn originates from Siberia while the *S. viminalis* parent is an interspecific cross between the male clone 78101 from Western Sweden and the female clone 78195 from southern Sweden. The parental clones of S_1_ were selected based on variation in phenology traits [42]. The pedigree is conserved in an orchard near to Uppsala (59°49′ N 17°40′ E, central Sweden) where all the plant material used in this study was collected.

#### Experiment 1 - Growth cessation and bud burst indoors

In spring 2008, growth cessation was assessed in the S_1_ pedigree in a phytotron experiment under controlled day length and temperature conditions. The two parental genotypes and 294 randomly drawn genotypes were propagated by means of hardwood cuttings and planted in 1.1 litre pots filled with Weibulls ‘Kron Mull’ (organic matter 95%; pH 5·5–6·5; 180 g m^-3^ N, 110 g m^-3^ P, 195 g m^-3^ K, 260 g m^-3^ Mg, 100 g m^-3^ S, 2000 g m^-3^ Ca). All genotypes were assigned to each one of three walk-in growth chambers in a complete randomized block design, where each chamber represented a block with one replicate. Plants were grown for four weeks under 20°C constant temperature, 70% relative humidity and 20 h photoperiod (300 μmol PAR m^-2^ s^-1^). The day length was then reduced to 16 h for one week, to 14 h for another week, and then one hour per week down to 10 h. After the first two weeks of growth, the plants were pruned and only the main shoot was preserved for the experiment. Plant height, i.e. the length of the stem from the emerging point on the wooden cutting to the tip of the apex, was measured once a week during week three and four, and three times per week thereafter. A sigmoid curve on the form y=b/(1+a × e^(-k × x)^), where y is plant height and x is the number of days since the first measurement, was fitted to the data of each plant in order to estimate the end of elongation growth, i.e. the day on which the estimated plant height reached 95% of the final value. The date of growth cessation was expressed as the number of days since the start of the reduction of daylength.

After the simulated autumn, the plants were subjected to an artificial winter at 8°C constant temperature and 9 h photoperiod for 9 weeks. The plants were then cut back, leaving 5 cm stem. Bud burst was forced by keeping a constant temperature of 12°C for six weeks and 11 h photoperiod. During this time the plants were checked for bud burst daily or every second day, depending on the speed of the process. Bud burst was defined as stage 3 according to the phenological scale previously used by Weih [6]. Individual plants were recorded as flushing on the day when at least one bud reached stage 3. The date of bud burst was expressed for each plant as the number of days since the beginning of forcing.

#### Experiment 2 - Bud burst, growth cessation and leaf senescence in the field

In spring 2008, plants of 463 genotypes were planted in an experimental field in Pustnäs, south of Uppsala (59°48′ N, 17°39′E, 25 m), at a spacing of 130 × 50 cm (i.e., about 20,000 plants ha^– 1^), according to a randomized complete block design comprising six blocks with one plant per genotype in each. Two border rows were planted around the experiment to reduce marginal effects. The plants were obtained by 5 cm hardwood cuttings rooted in 0.5 L peat pots with Weibulls ‘Kron Mull’ as growing medium. The plants were grown for five weeks in a greenhouse and then transferred outside for hardening. Before planting, the site was appropriately prepared [62], including ploughing, harrowing and repeated application of a systemic herbicide (Glyphomax, Dow AgroSciences, Indianapolis, IN). The plantation was irrigated in summer 2008 and weed controlled during the whole experimental period. The plants were cut back in winter 2009 and fertilized in spring 2009, 2010 with N P K (21-4-7) corresponding to 80 kg N/ha and year.

Growth cessation as defined by shoot apex abscission was scored using the highest shoot of each plant in 2008 and 2009 from the end of August and once to twice per week depending on the rate of progression. Leaf senescence and abscission was visually estimated on October 31 2009 and 2010 according to the following leaf senescence index (LSI): 0 = no leaves left on the plant (100% abscission); 0.5 = less than 10% brownish leaves (~ 95% abscission); 1= 10 to 20% brownish leaves (~ 85% abscission); 1.5 = 20 to 30% brownish or yellow leaves (~75% abscission); 2 = 30 to 40% yellow leaves (~65% abscission); 2.5 = 40 to 50 yellow and green leaves (~55% abscission); 3 = 50 to 65 green leaves (~40% abscission); 3.5 = 65 to 80% green leaves (~30% abscission); 4=more than 80% green leaves (~10% abscission). Bud burst, defined as in Experiment 1, was assessed twice a week during April and May 2009 and 2010. Date of bud burst and date of apex abscission were expressed as day of the year (DOY), i.e. number of days since January 1. Plants were cut back in January 2009. Therefore spring phenology was assessed on stumps in 2009 and on one-year old shoots in 2010; and autumn phenology was assessed on one-year shoots in 2008 and 2009, and on two-year old shoots in 2010. In order to characterize the weather conditions at the plantation site, temperature sum (cumulated mean temperatures CMT,°C), number of chill days with mean temperature below 5°C (CD<5°C), and the number of days without rain (cumulated dry days, CMDD) were calculated in relevant periods of time in all years of study from the records of a nearby meteorological station (Figure 6).

### Statistical analyses and QTL mapping

The complete set of data from each experiment, which included all genotypes that had records for at least three ramets, was analysed with a mixed model ANOVA and variance components analysis to determine the effects of genotype (set as random factor) and block (set as fixed factor), and to estimate the broad-sense heritability, i.e. the ratio between genetic variance and total phenotypic variance. The genotype × block interactions in the field were tested using grouped data from two years. Subsequent analyses were performed on unadjusted mean values among blocks in each experiment. Correlations among genotype means were calculated and plotted (Additional file 1: Figure S1) for all combinations of traits in each experiment and across experiments. The year effect and its interaction with genotype were tested in a separate ANOVA for all the traits assessed in the field. QTL analyses were performed with MapQTL ® 6.0 [63] and the linkage map previously developed for the *Salix* pedigree S_1_ was used [35]. First, interval mapping [64] was applied using 1.0 cM steps across the genome to determine putative QTLs involved in the variation of each trait. Following Churchill and Doerge [65], the logarithm of the odds (LOD) threshold for QTL significance was empirically estimated from 5,000 permutations of phenotypic data. Two theoretical critical thresholds were considered for detection of a putative QTL: the first corresponding to genome wide error rate of 5% was used to define significant QTLs, and the second corresponding to a type I error of 5% at the chromosome level was used to define suggestive QTLs (‘suggestive linkage’, [66,67]. Multiple QTL mapping (MQM) [68] was then performed on the same data: the nearest SNP marker to each putative QTL peak was used as a cofactor to control the genetic background while testing at a position in the genome. Only markers close to QTLs significant at the genome wide level were used as cofactors in MQM. When a cofactor was also a flanking marker of the tested region, it was automatically excluded from the model. The number of cofactors used varied between 1 and 5. A 1.5-unit drop off on either side of the local LOD score peak was used to determine heuristic support intervals for significant QTLs.

A backward selection procedure, at a test level of 0.05, was performed to determine whether a particular QTL could be dropped from the model resulted from the MQM analysis. For each trait, the model involved the genotype at the closest marker to the corresponding putative QTL. Pairwise epistatic interactions between all putative QTLs were tested, via the corresponding marker × marker interaction treated as random effects, by comparing the deviance of a model including all main effect QTLs and the specific epistatic interaction, with the deviance of a model including only the main effect QTLs and no epistatic interaction [69]. The deviance test (p ≤ 0.001) was applied for testing interactions. The contribution of each significant QTL and epistatic interaction was then estimated by maximum-likelihood variance components analysis. Statistical analyses were performed using R [70] and SPSS v.19.

### Improved mapping of two QTL hot spots for phenology on LG II and X

Genomic DNA from 463 genotypes from the S_1_ pedigree was extracted as in Berlin et al. (2010). Two chromosomal regions on LG II (75–90 cM) and LG X (84–93 cM ) containing QTLs for all traits except bud burst in the field 2010 were selected to increase the marker density and new primer pairs were designed in these regions (Additional file 4: Methods and Additional file 5: Table S3). Gene segments were amplified by PCR and sequenced in each parent as described in Berlin *et al*. [35]. A total of 96 SNPs were selected for genotyping, 59 from LG II region and 37 from LG X region. Linkage maps were constructed as in Berlin et al. [35], for details see Additional file 4: Methods.

New QTL analyses for group II and X with the denser linkage maps were conducted. In the linkage map used for the QTL analysis all AFLP markers were removed since only 96 out of 463 individuals were genotyped with AFLPs and thus did not add much information to the analysis. When several markers were located at the same position only the most informative was kept. MQM mapping using MapQTL ® 6.0 [63] was conducted for comparison of QTLs between new and earlier linkage maps. The most informative marker close to the peak position of the QTL was used as cofactor in the MQM analysis.

### Comparative mapping and in-silico selection of candidate genes

Physical coordinates of the QTLs were obtained from anchored markers in the *P. trichocarpa* genome assembly version 3 (http://www.phytozome.net/poplar). For each QTL interval, positions of sequences containing the SNP markers flanking 1.5-LOD on both sides of the LOD score peaks were determined by BLASTN searches. Once the regions were determined, gene models (predicted by the Gnomon gene prediction tool) were downloaded using BioMart that were subsequently annotated using the *P. trichocarpa* version 3 annotation information. Annotated gene models positioned in QTLs were searched for putative candidate genes. Since growth cessation and bud set are mainly controlled by photoperiod, genes in the photoperiod pathway and the circadian clock were considered as candidate genes for these traits (Additional file 3: Table S2). Genes controlling budburst and leaf senescence are less well known and candidate genes for those traits with functional characterization are scarce or absent. We therefore did not attempt to identify any such candidate genes.

## Competing interests

The authors declare that they have no competing interests.

## Authors’ contributions

LG performed phenotypic measurements, conducted and interpreted the phenotypic and QTL analyses and wrote the manuscript. SB created the new linkage map, performed the genome analysis and wrote the manuscript. MW read and commented on the manuscript and provided funding for measurements in the field experiment. NG and UL were involved in phenotypic measurements, selected candidate genes, read and commented on the manuscript. ACRW conceived the study, performed phenotypic measurements, conducted QTL analysis with the new linkage map, wrote the manuscript and provided funding for the project. All authors read and approved the final manuscript.

## Supplementary Material

Additional file 1: Figure S1 Pairwise plots of phenology traits across years and environments based on mean values for each individual in the mapping population S1. Red line show the linear fit of points. Click here for file

Additional file 2: Table S1 Genomic regions for each QTL. Click here for file

Additional file 3: Table S2 Candidate genes within QTL regions for each trait. Positions of the genes are indicated in Figures 2 and 3. Click here for file

Additional file 4: Methods Improved linkage map in two QTL hot spots for phenology on LG II and X. Click here for file

Additional file 5: Table S3 Markers in fine mapping areas. Click here for file
