# Supplementary material for: Genetic architecture of spring and autumn phenology in Salix
Source: BMC Plant Biol. 2014 Jan 17;14:31. doi: 10.1186/1471-2229-14-31 (PMC3945485; doi:10.1186/1471-2229-14-31)
Supplement: Additional file 1: Figure S1 — Pairwise plots of phenology traits across years and environments based on mean values for each individual in the mapping population S1. Red line show the linear fit of points. [file 1471-2229-14-31-S1.pptx]

## Slide 1
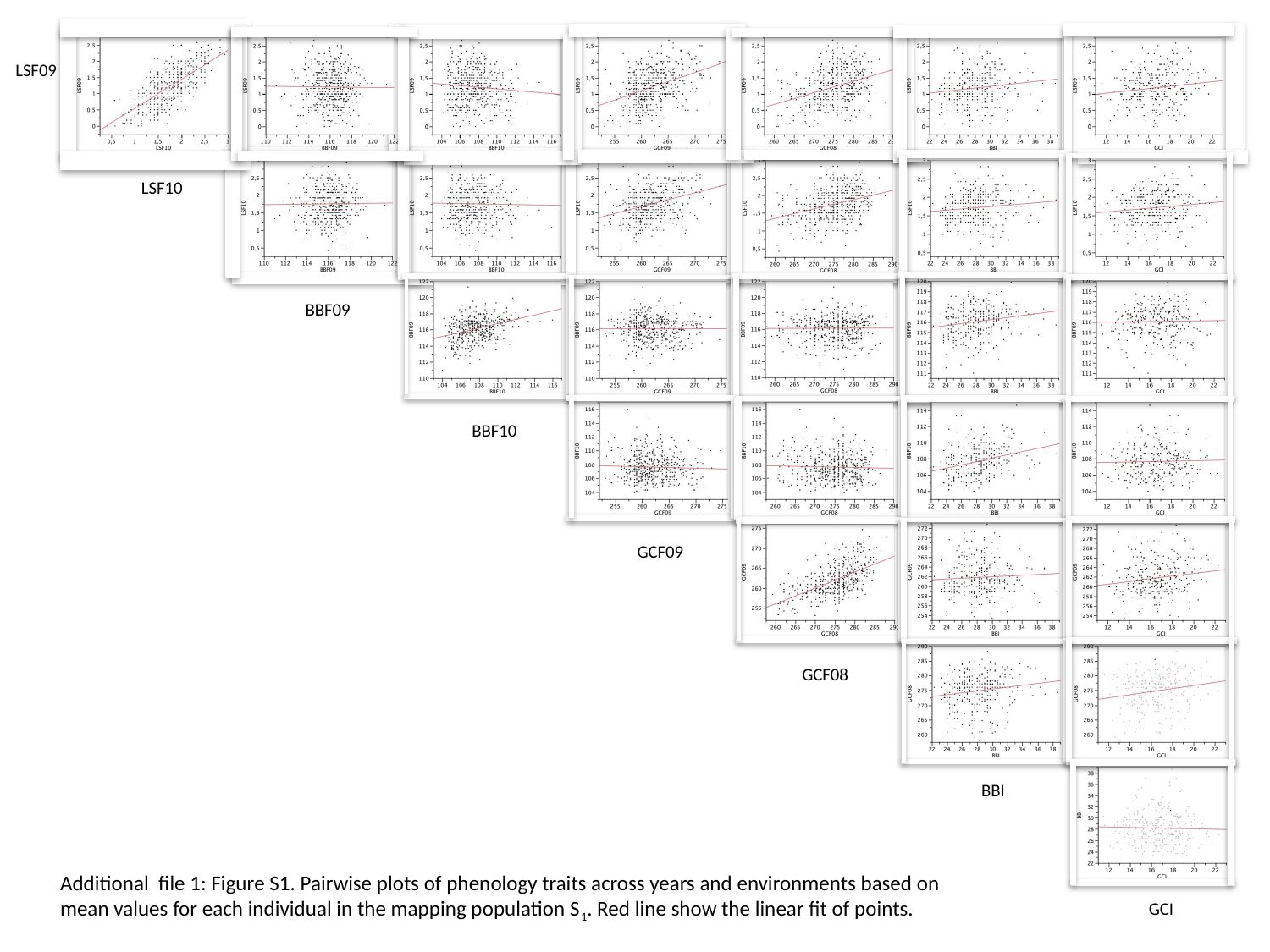

LSF09
LSF10
BBF09
BBF10
GCF09
GCF08
BBI
GCI
Additional file 1: Figure S1. Pairwise plots of phenology traits across years and environments based on mean values for each individual in the mapping population S1. Red line show the linear fit of points.
