# Supplementary material for: Genetic architecture of spring and autumn phenology in Salix
Source: BMC Plant Biol. 2014 Jan 17;14:31. doi: 10.1186/1471-2229-14-31 (PMC3945485; doi:10.1186/1471-2229-14-31)
Supplement: Additional file 4: Methods — Improved linkage map in two QTL hot spots for phenology on LG II and X. [file 1471-2229-14-31-S4.docx]

Additional file 4: Methods.

*Improved linkage map in two QTL hot spots for phenology on LG II and X*

A perl script was developed for automatic design of primers evenly spaced in the QTL regions (Table S1) using the Populus trichocarpa genome sequence v 1.1 as template (http://www.jgi.doe.gov/genome-projects/). A total of 167 primer pairs (named II-f1 to II-f180) were positioned in LG II region and 80 primer pairs were positioned in LG X region (named X-f1 to X-f80). The parents were PCR amplified and sequenced for all gene segments. The whole mapping population was genotyped for 96 polymorphic SNP markers of which 70 were located in different genes, thus in 26 gene segments, two SNPs were positioned. In that case, the SNPs were named e.g. II_f20 and II_f20_2 (Table S1). The SNPs were genotyped using Illumina’s Golden Gate Assay ([Fan *et al.*, 2003](#_ENREF_13)) at the SNP & SEQ Technology Platform, Uppsala University. Linkage maps were constructed as in Berlin *et al*. ([2010](#_ENREF_3)). Out of the 96 additional SNPs that were selected for genotyping of the hot spot regions, 90 SNPs resulted in usable genotype data (Table S1). The genotypes from the new 90 SNPs were added to the genotypes from the 639 markers that previously were used to build the S_1_ linkage map in Berlin et al. ([2010](#_ENREF_3)) and new consensus linkage maps were constructed using all 729 markers. Of the 54 new markers designed to map at LG II, 49 grouped together with earlier markers into one main group of 145.7 cM and five new markers made up a separate group of 27.8 cM. The genetic distance of the hot spot region of the main group was 43.9 cM and the total number of SNP markers was 61 (47 new and 14 old markers) The new marker density is thus 1.4 marker per cM in LG II region. Within the LG II region a 0.8 Mbp inversion between markers II_f68 and II_f79 when comparing Populus and Salix LG II was detected. Two new LG II markers mapped on other LGs; II_f60 mapped on LG V and II_f76 on LG XIV. The 36 new LG X markers form two groups, one large making up 144.2 cM and a doublet group of 1.4 cM with one old marker. The number of markers in the hot spot region has increased to 40 markers of which 35 were designed for the purpose of this study. The resulting marker density is 1.8 markers per cM in LG X region.
